# Soft-tissue and dermal arrangement in the wing of an Early Cretaceous bird: Implications for the evolution of avian flight

**DOI:** 10.1038/srep14864

**Published:** 2015-10-06

**Authors:** Guillermo Navalón, Jesús Marugán-Lobón, Luis M. Chiappe, José Luis Sanz, Ángela D. Buscalioni

**Affiliations:** 1 School of Earth Sciences, University of Bristol, Wills Memorial Building, Queens Road, Bristol, BS8 1RJ, UK; 2 Unidad de Paleontología, Facultad de Ciencias, Universidad Autónoma de Madrid, Campus de Cantoblanco, Madrid, Spain.; 3 Dinosaur Institute, Natural History Museum of Los Angeles, 900 Exposition Boulevard, Los Angeles, CA 90007, USA

## Abstract

Despite a wealth of fossils of Mesozoic birds revealing evidence of plumage and other soft-tissue structures, the epidermal and dermal anatomy of their wing’s patagia remain largely unknown. We describe a distal forelimb of an enantiornithine bird from the Lower Cretaceous limestones of Las Hoyas, Spain, which reveals the overall morphology of the integument of the wing and other connective structures associated with the insertion of flight feathers. The integumentary anatomy, and myological and arthrological organization of the new fossil is remarkably similar to that of modern birds, in which a system of small muscles, tendons and ligaments attaches to the follicles of the remigial feathers and maintains the functional integrity of the wing during flight. The new fossil documents the oldest known occurrence of connective tissues in association with the flight feathers of birds. Furthermore, the presence of an essentially modern connective arrangement in the wing of enantiornithines supports the interpretation of these primitive birds as competent fliers.

Exceptional fossil preservation allows access to crucial information regarding the integumentary structures^1^, internal organs^2^, and other soft-tissues^3^ of early birds. These rare occurrences have proven crucial for a better understanding of the origin and early evolution of avian physiology^2^,^4^,^5^, behavior^4^,^5^, and particularly, the characteristics of their flight^4^,^5^,^6^. The anatomy of the connective tissue of the forelimb of basal birds, however, is largely unknown beyond general observations (i.e., evidence of propatagium^7^,^8^). Nonetheless, the characteristics of the wing’s connective tissue are central to flight mechanics given that muscles, tendons, ligaments, and conjunctive tissues connect anatomically the skeletal forelimb with an airfoil composed almost entirely of feathers^9^,^10^. Here we describe a new fossil (MCCMLH31444; Museo de las Ciencias de Castilla-La Mancha, Cuenca, Spain) (Fig. 1) of an upper Barremian (~125 MYa) bird from the Las Hoyas Konservat-Lagerstätten^11^,^12^. Consisting of the distal portion of a left wing, MCCMLH31444 displays a metacarpal III projecting distally further than metacarpal II (following the digit identity formula of the avian hand as I–II–III^13^), a synapomorphy of Enantiornithes, and an overall morphology consistent with the anatomy of this clade^14^. The fossil preserves the anatomy of the wing’s patagia^10^ and different connective structures associated with the attachment of the remigial feathers^9^ in exceptional detail.

## Results

The distal wing of MCCMLH31444 is likely exposed in ventral view, as evidenced by the presence of ventral flexor depressions in the exposed side of the pre-ungual phalanges of digit I and II and the metacarpal II^15^,^16^. The preserved portions of the ulna, radius, and metacarpals, and the complete manual digits, are all surrounded by abundant remains of epidermal and dermal connective tissues in close association with plumage. Eight to nine large and strongly asymmetrical primaries, 10–12 secondaries, and the remains of plumulaceous coverts are preserved as carbonized keratinous structures. A halo of brownish-to-yellowish epidermic and dermic tissue outlines the skeletal elements of the wing except for the ungual phalanges of digits I and II and their keratinous sheaths. The connective tissue associated with the plumage shows the same post-mortem folding of the skeletal elements, indicating that these soft tissues are preserved in anatomical position.

**Figure 1:**
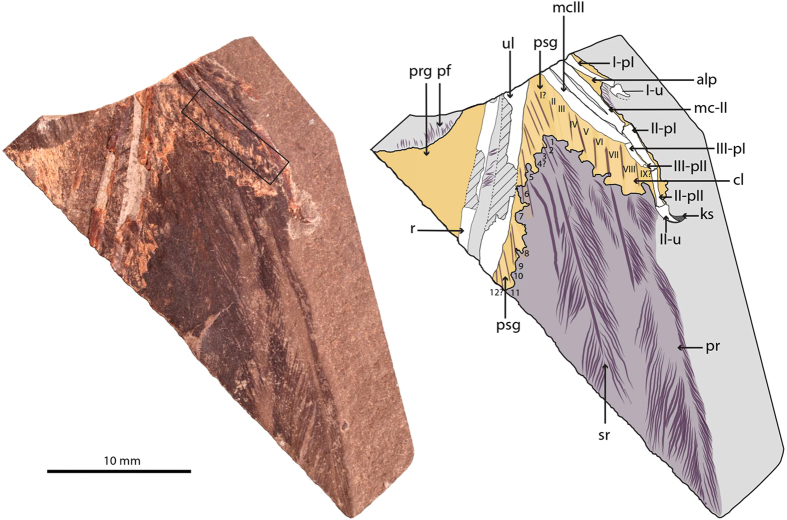
MCCMLH31444, enantiornithine bird (Lower Cretaceous, Las Hoyas, Spain). Photograph and interpretive drawing of slab. Black-lined inset in the photograph delineates the area of the transition between bone and soft tissue magnified in Fig. 2A. Abbreviations: alp, alular patagium; cl, calamus; ks, keratinous sheath; mc-II; metacarpal II; mc-III, metacarpal III; pf, plumulaceous feathers; pr, primary remex; prg, propatagium; psg, postpatagium; r, radius; sr, secondary remex; ul, ulna; I-pI, first phalanx of digit I; I-u, ungual phalanx of digit I; II-pI, first phalanx of digit II; II-pII, second phalanx of digit II; II-u, ungual phalanx of digit II; III-pI, first phalanx of digit III; III-pII, second phalanx of digit III.

Three epidermal structures (i.e., patagia^10^) are noticeable in MCCMLH31444: the propatagium, the alular patagium, and the postpatagium (Fig. 1). The morphology of the propatagium is remarkably similar to that of modern birds^10^,^17^, other enantiornithines^7^, and the Chinese Early Cretaceous *Confuciusornis sanctus*^8^, in which this large epidermal fold connects the wrist to the shoulder (Fig. 1). The propatagium shows a scattered pattern of feather follicles distributed across its entire surface (Suplementary Fig. S1, online). The alular patagium extends from the cranial surface of the midshaft of the metacarpal II to almost the caudodistal end of the first phalanx of digit I (Fig. 1), thus showing a similar distribution to that of most modern birds^10^,^17^. Particularly remarkable is the presence of an almost completely preserved postpatagium, extending from the caudoproximal base of the ungual phalanx of digit II to the preserved proximal-most caudal portion of the ulna and in clear association with the wing’s plumage (Fig. 1). The preserved calami of flight feathers (i.e., primaries and secondaries) are embedded in the postpatagium, forming the same sinuous caudal outline visible in the naked (i.e., unfeathered) wing of modern birds^10^,^17^ (Fig. 1). Also, the reduced digit III appears to be completely lodged in the postpatagium, as in modern birds^10^,^17^ (Fig. 1), thus confirming previous claims that in Enantiornithes this reduced digit was attached to the digit II by soft-tissue structures^18^.

**Figure 2:**
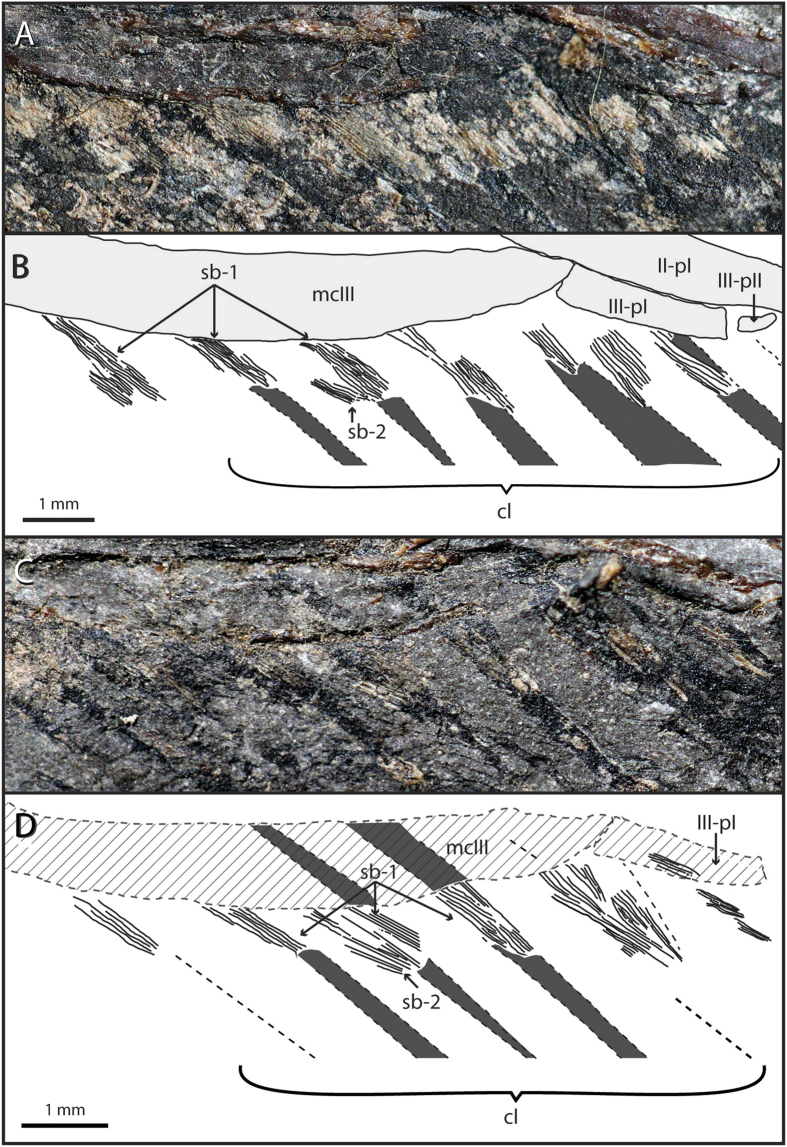
Arrangement of striated bundles in the manual region of postpatagium. (**A**,**B**) Photograph and interpretive drawing of tissue and details (Type 1 and 2 bundles of stripes) below skin level of the postpatagium (inset shown in Fig. 1). (**C**,**D**) Photograph and interpretive drawing of the same region in the counterslab. Abbreviations: cl, calami; mcIII, metacarpal III; sb-1, Type 1 striated bundle; sb-2, Type 2 striated bundle; II-pI, first phalanx of digit II; III-pI, first phalanx of digit III; III-pII, second phalanx of digit III. (Photograph by José Antonio P. Gracia).

**Figure 3:**
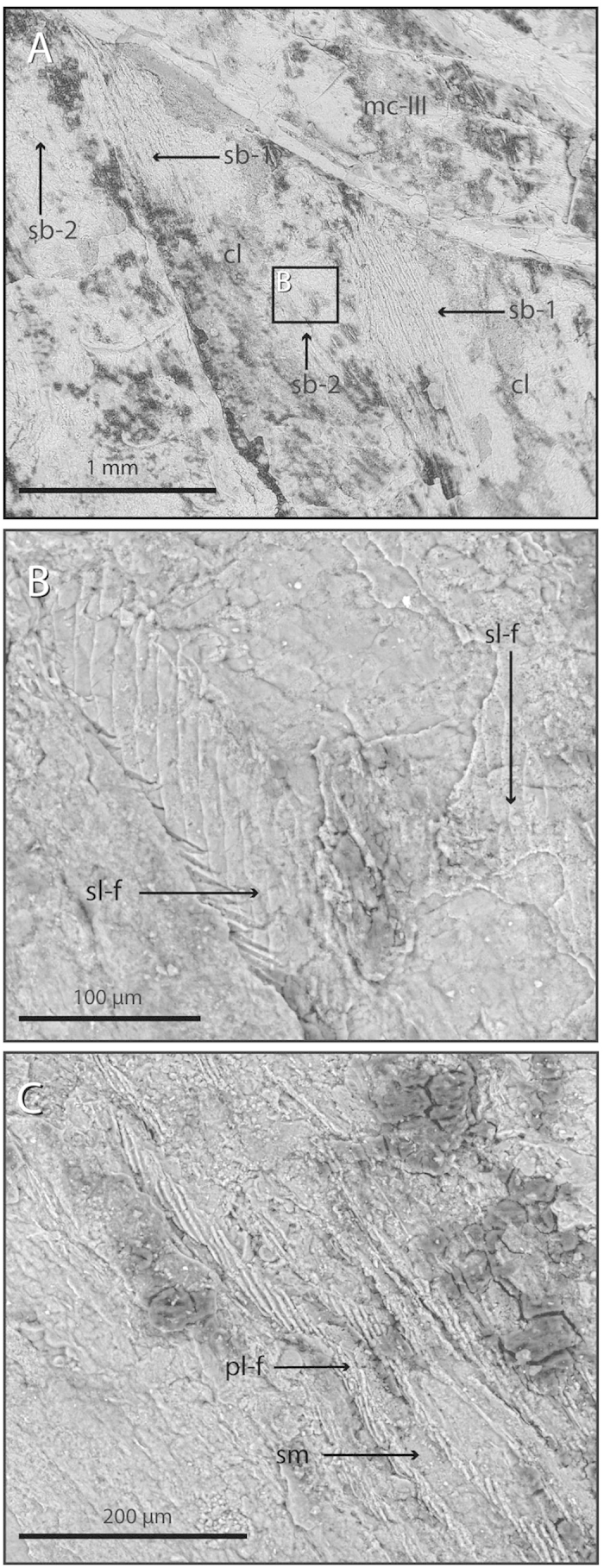
Microstructure of striated bundles. (**A**) SEM image of two consecutive primary feather calami and their respective Type 1 and Type 2 striated bundles. (**B**) SEM close-up image of the area delineated by an inset in (**A**), of a Type 2 striated bundle containing strap-like fibers. (**C**) SEM close-up of a Type 1 striated bundle showing its alternating pattern of parallel plait-like fibers and smooth matrix. Abbreviations: cl, calami; mcIII, metacarpal III; sb-1, type 1 striated bundle; sb-2, type 2 striated bundle; pl-f, plait-like fibers; sm, smooth matrix; sl-f, strap-like fibers.

The connective tissue surrounding the proximal-most portion of the calami of the remiges is characterized by a serial striation pattern (Fig. 2). The striations are arranged in discrete and truncated cone-shaped to ribbon-shaped bundles that attach diagonally to the medial portion of the calamus of each feather (Fig. 2). In some of the best-preserved regions, it is possible to identify several striated bundles of fibers that attach to the calamus at different angles and one above the other (Figs 2 and 3A). In the proximal-most region of each bundle—opposite to their feather attachment—the striation lines are more tightly joined together, slightly curving with respect to the main orientation of the bundle (Fig. 2).

SEM inspection reveals that the majority of these striated bundles are composed of plait-like fibers arranged in parallel and alternating with smooth regions of subequal width (here label as Type 1 striated bundles, Fig. 3A,C). The plait-like fibers are made of smaller fibers rolled in a helical pattern (Fig. 3C); the size range and arrangement of these smaller structures is congruent with their interpretation as fibrous collagenous fibers^19^. Additionally, we label as Type 2 some of the striated bundles in the postpatagium containing strap-like fibers. These are flat and wider than the plait-like ones, and are patterned with diagonal striations with respect to the main axis of the fibers (Fig. 3B). These strap-like fibers are only visible in two of the best-preserved striated bundles and they are associated diagonally with the calami at a higher angle than the plait-like fibers that compose the Type 1 striated bundles (Figs 2 and 3).

**Figure 4:**
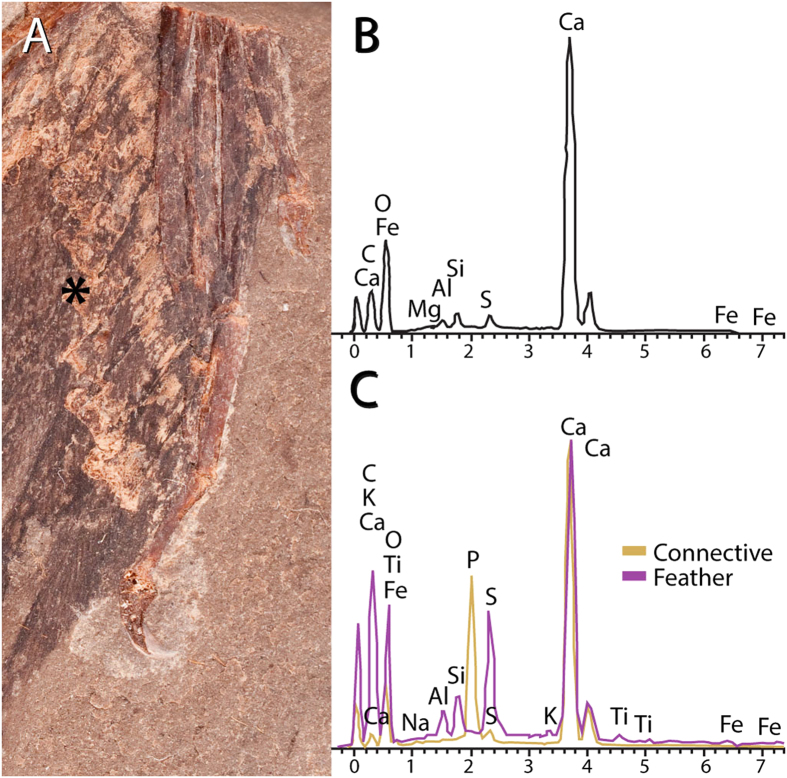
EDAX data from different regions of the slab of MCCMLH31444. (**A**) Photograph of part of MCCMLH31444 indicating the region (asterisk) where the EDAX analysis was performed (over the remains of connective tissue). (**B**) EDAX profile of matrix, primarily showing a high abundance of Ca as expected for a limestone locality. (**C**) EDAX profile of feathers (purple profile) and connective tissues (brownish). Feathers are composed by higher concentrations of K, O, S, C and Fe as well as traces of Ti; connective tissues show a remarkably different composition from both the matrix and the fossil feathers, with a much more flat profile and a higher peak of P and Ca, evidencing the underlying calcium phosphatization process. One sample was collected from each region.

Element analyses (EDAX) indicate that the connective tissues of MCCMLH31444 are mainly preserved as calcium phosphate while the plumage has likely undergone a carbonization process (Fig. 4). Phosphatization of soft tissues is generally associated with changes in pH levels and conditions that allow steady mineral precipitation^20^. These decay conditions fit the combination of taphonomic factors—obruption, stagnation, and bacterial sealing—proposed as responsible for the exceptional preservation of the Konservat Lagerstätte of Las Hoyas^11^.

## Discussion

The parallel pattern of plait-like fibers alternating with an unorganized matrix in MCCMLH31444 is characteristic of modern tendons and ligaments that are subject to multidirectional mechanical stresses^19^,^21^. Even though the structural composition of modern tendons and ligaments is highly variable depending on their size and mechanical requirements^19^,^21^, the strap-like fibers also preserved in the new fossil also show an overall morphology similar to that of structures interpreted as fossilized muscle tissues in theropods^22^,^23^. The general morphology and arrangement of the system of ligaments, elastic tendons, and smooth muscles associated with the follicles of flight feathers in the postpatagium of MCCMLH31444 is strikingly similar to that present in living birds^9^,^10^, strongly suggesting that a comparably elaborate dermal system is preserved in the new fossil.

In modern birds, networks of smooth muscles attached by elastic tendons to the outer walls of feather follicles control the coordinated movement of groups of feathers^9^,^10^,^24^,^25^. In the wing, a well-developed array of such tendons and feather muscles—erectors, depressors, retractors and rotators^9^—together with other ligamental structures (i.e., *Ligg. phalangoremigalia distalia*, *digitationes remigiales*^10^) bestows structural strength and allows the range of movement crucial for controlling lift and maneuverability during flight^9^,^25^. For instance, the contraction of rotators results in a counter-clockwise movement of the flight feathers^9^,^25^, which counteracts the passive rotation of the feathers caused by aerodynamic stresses and impacting the wing’s ability to generate lift^9^,^25^. The comparable arrangement and microstructure of the plait-like muscle fibers observed in MCCMLH31444 suggest that they were capable of providing structural support and buffer against multidirectional forces, such as the ones exerted on the remigial feathers during flight in modern birds. This overall similarity suggests that the wing of MCCMLH31444, and most likely that of other flighted enantiornithines, formed an airfoil capable of enduring important aerodynamic stresses.

The evolution of the modern network of smooth muscles, elastic tendons and ligaments involved in the function of the wing’s flight feathers was a paramount event in the fine-tuning of aerodynamic capabilities in birds. By allowing these feathers to be repositioned in tandem, this sophisticated dermal system helped the wing to maneuver as a single functional unit and to cope with the strenuous aerodynamic stresses of flapping flight. The 125-million-year-old MCCMLH31444 provides the oldest reported evidence of this intricate connective network and its earliest phylogenetic occurrence. The remarkably modern anatomy and arrangement of the connective tissues preserved in the wing of MCCMLH31444 implies that Early Cretaceous enantiornithines had already developed forelimbs morphologically well adapted for flight, losing most of the primitive grasping functions attributed to the dinosaurian forerunners of birds^5^.

Although still showing a suite of primitive skeletal traits^4^,^14^, even the earliest enantiornithines (i.e., *Protopteryx fengningensis*^26^) had already developed forelimb elements of modern proportions, a carinate sternum, and an advanced pectoral girdle including a triosseal canal for the passage of flight muscles^4^,^14^, all of which suggest active flapping flight and a wing stroke similar to that of present-day birds^4^,^14^. Integumentary similarities with modern birds, such as wings with identical feather arrangement and a well-developed alula (i.e., bastard wing)^18^,^27^, also point to the same functional conclusions^4^,^18^,^27^. The preservation of three important patagia—propatagium, alular patagium, and postpatagium—together with the above-mentioned dermal system in the wing of MCCMLH31444, an Early Cretaceous enantiornithine, lends strong support to the notion that these primitive avians had achieved aerodynamic competence comparable to those of many modern birds.

## Additional Information

**How to cite this article**: Navalón, G. *et al.* Soft-tissue and dermal arrangement in the wing of an Early Cretaceous bird: Implications for the evolution of avian flight. *Sci. Rep.* **5**, 14864; doi: 10.1038/srep14864 (2015).

## Supplementary Material

Supplementary Information
