# Supplementary material for: Soft-tissue and dermal arrangement in the wing of an Early Cretaceous bird: Implications for the evolution of avian flight
Source: Sci Rep. 2015 Oct 6;5:14864. doi: 10.1038/srep14864 (PMC4594305; doi:10.1038/srep14864)
Supplement: Supplementary Information [file srep14864-s1.pdf]

Soft-tissue and dermal arrangement in the wing of an Early Cretaceous bird: Implications for the evolution of avian flight

Guillermo Navalón[1,2], Jesús Marugán-Lobón[2,3], Luis M. Chiappe[3*], José Luis Sanz[2], Ángela D. Buscalioni[2]

1 School of Earth Sciences, University of Bristol, Wills Memorial Building, Queens Road, Bristol, BS8 1RJ, UK.

2 Unidad de Paleontología, Facultad de Ciencias, Universidad Autónoma de Madrid, Campus de Cantoblanco, Madrid, Spain.

3 Dinosaur Institute, Natural History Museum of Los Angeles, 900 Exposition Boulevard, Los Angeles, CA 90007, U.S.A.

*corresponding author: lchiappe@nhm.org

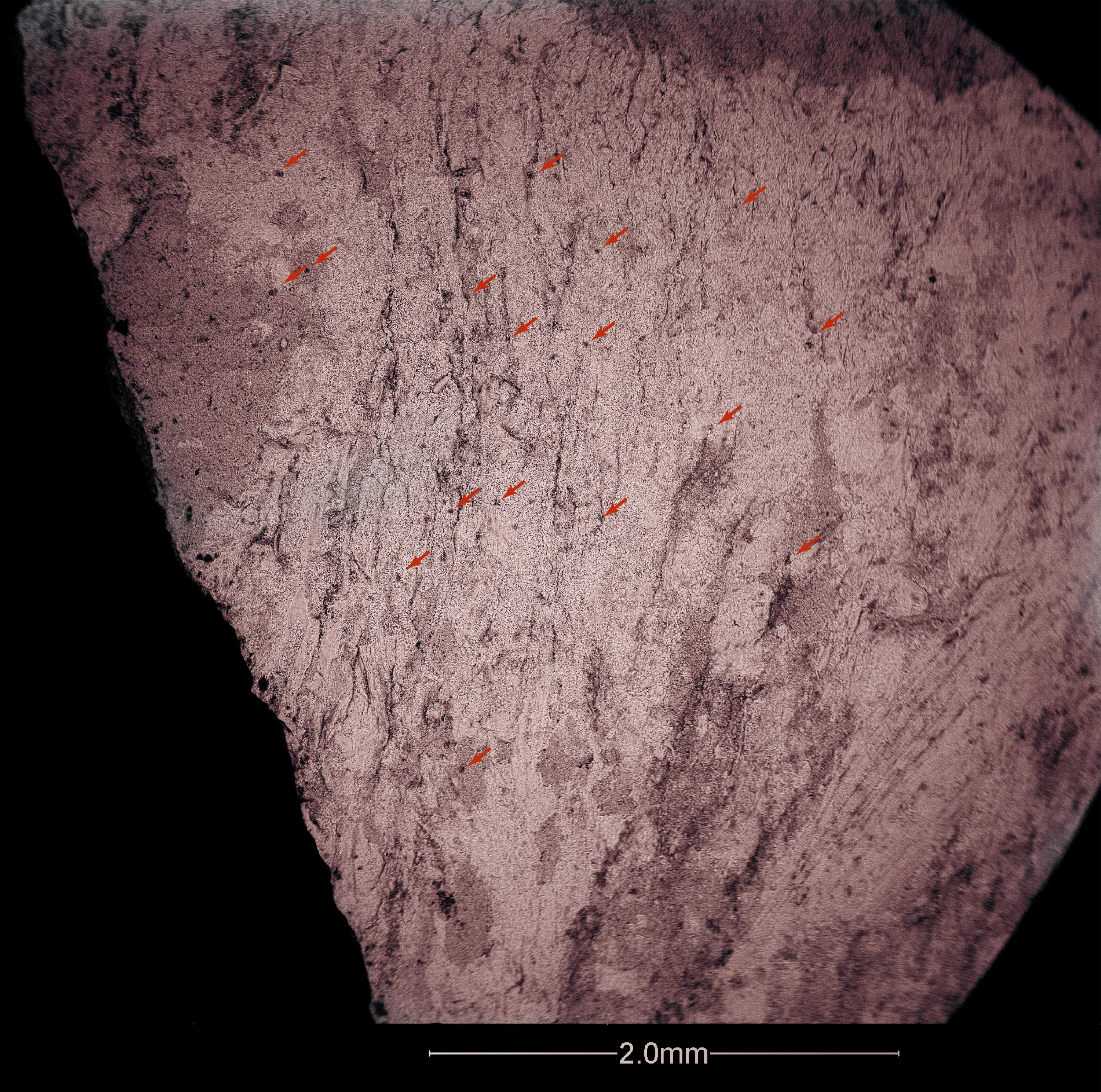
2.0mm

Figure S1. Scattered pattern of follicles in the propatagium of MCCMLH31444 (SEM). Red arrows indicate the location of some follicles.
